# Intravascular Leiomyomatosis as a Rare Cause of Nonthrombotic Pulmonary Embolism

**DOI:** 10.1155/2020/6084061

**Published:** 2020-07-14

**Authors:** Julie Van Maercke, Anne-Sophie Van Rompuy, Willy Poppe, Tom Verbelen, Marion Delcroix, Catharina Belge

**Affiliations:** ^1^Department of Respiratory Diseases, KU Leuven-University of Leuven and University Hospitals Leuven, Leuven, Belgium; ^2^Department of Anatomical Pathology, KU Leuven-University of Leuven and University Hospitals Leuven, Leuven, Belgium; ^3^Department of Gynecology, KU Leuven-University of Leuven and University Hospitals Leuven, Leuven, Belgium; ^4^Department of Cardiac Surgery, KU Leuven-University of Leuven and University Hospitals Leuven, Leuven, Belgium

## Abstract

Intravascular leiomyomatosis (IVL) is a very rare condition. It is characterized by the proliferation of benign smooth muscle cells within vascular structures without invasion of these tissues. Symptoms depend on the site of origin and the extent of invasion. Rarely, this neoplasm is located in the inferior vena cava or in the pulmonary vasculature potentially causing symptoms of dyspnea, chest pain, or syncope. We report the case of a 53-year-old woman who was referred to our hospital with extensive pulmonary embolism comprising of a subtotal occlusion of the right pulmonary artery with extension into the left pulmonary artery. Due to persistent dyspnea (New York Heart Association class II) despite anticoagulation, after a six-week period, imaging was repeated and showed stable findings. As she was not responding to adequate anticoagulant therapy, intima sarcoma of the pulmonary artery was suspected, and a pulmonary endarterectomy (PEA) was performed. A smooth, white, intravascular mass was easily and completely removed. Analysis demonstrated a lesion consisting of cells without atypia, showing expression of alpha-smooth muscle actin (alpha SMA) and desmin with partial expression of estrogen receptor (ER) and progesterone receptor (PR), leading to the diagnosis of intravascular leiomyomatosis. The patient fully recovered. Complete surgical removal of the intravascular tumor is recommended to relieve symptoms and prevent possible complications. Clinicians have to be aware that in unresolved pulmonary embolism, nonthrombotic and rare causes, like an intima sarcoma or intravascular leiomyomatosis, should be considered.

## 1. Introduction

Pulmonary embolism is a common and potentially life-threatening medical condition. It is usually caused by a deep vein thrombosis [1]. Other causes of intravascular obstruction are described [2] but are extremely rare. This causes substantial delay in correct diagnosis and treatment, which is often only obtained by a multidisciplinary approach. We present a case of an extensive pulmonary embolism not responding to anticoagulation therapy, which revealed to be due to intravascular leiomyomatosis.

## 2. Case Presentation

**Figure 1:**
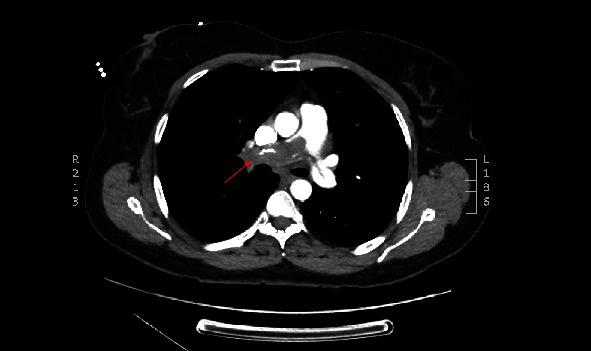
Transverse view of contrast-enhanced computed tomography images demonstrating a pulmonary embolism with subocclusion of the right pulmonary artery with extension to the left pulmonary artery and truncus pulmonalis.

**Figure 2:**
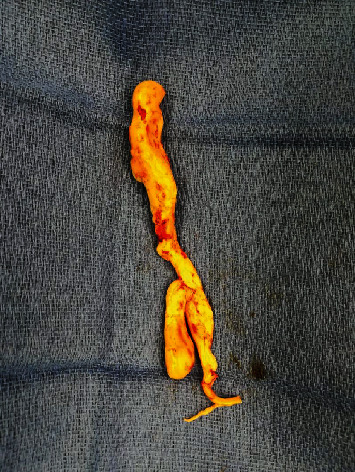
Macroscopic view of the resection specimen with a length of 15 cm and a smooth surface (“wormlike” appearance).

**Figure 3:**
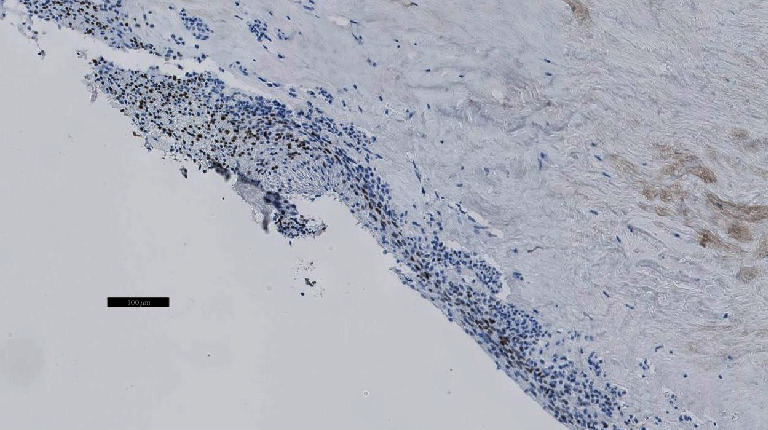
Immunohistochemical examination of the tumor (enlargement ×10) shows benign-appearing smooth muscle cells with expression of estrogen receptor (ER), marked in brown.

A 53-year-old woman was referred to the cardiology department in an affiliated center suffering from dyspnea during exercise (New York Heart Association class II) which she had experienced for a three-week period. She had recently taken a two-hour flight. She had no past medical problems and no family history of cardiovascular or lung diseases. She did not take any medication. Her physical examination was unremarkable. Echocardiography showed a normal systolic and diastolic function with a systolic pulmonary artery pressure (PAPsys) of 33 mmHg. Blood count was normal besides a slightly elevated D-dimer test of 560 *μ*g/L (0-500 *μ*g/L). Computed tomography (CT) showed a subtotal occlusion of the right pulmonary artery and extension into the left pulmonary artery (Figure 1). Based on a diagnosis of acute pulmonary embolism, anticoagulant therapy with low molecular weight heparin (LMWH) was initiated. Although not recommended by the guidelines, screening for underlying malignancy by positron emission tomography- (PET-) CT scan was performed, which only exposed a fibromatous uterus with thrombosis of the right ovarian vein. The patient was discharged with apixaban five milligram twice daily. Due to persistent dyspnea, imaging was repeated after six weeks, showing no regression of the pulmonary embolism. As a result of this unusual outcome, she was referred to our center for treatment of chronic thromboembolic pulmonary hypertension. Based on the CT aspect with extensive proximal material in the absence of pulmonary hypertension, a nonthrombotic cause of pulmonary obstruction was suspected. Histopathological analysis of a biopsy obtained by right heart catheterization showed no microscopic or immunohistochemical evidence of an intima sarcoma. To obtain further diagnosis and treatment, a pulmonary endarterectomy (PEA) was performed. During this procedure, a smooth, white, intravascular mass was visualized (Figure 2) which was easily resected in its entirety. Macroscopic inspection of the pulmonary artery was normal. Pathological analysis of the specimen demonstrated a lesion consisting of spindle cells without atypia, showing expression of alpha SMA and desmin with partial expression of estrogen and progestogen receptors (Figure 3). Those findings led to the diagnosis of intravascular leiomyomatosis (IVL) without malignant transformation. The patient recovered completely. CT imaging of the thorax revealed no residual intravascular filling defects. Subsequently, our patient was referred to the department of gynecology. Magnetic resonance imaging (MRI) of the pelvis confirmed the presence of a fibromatous uterus without signs of malignancy. Given the postmenopausal state of the patient without strong evidence of intracaval tumor thrombosis, the gynaecologists forwent a hysterectomy. Follow-up with CT imaging of the thorax and abdomen is planned after one year.

## 3. Discussion

Pulmonary embolism is a potentially life-threatening medical condition; most commonly caused by a deep vein thrombosis [1]. However, fat embolism, amniotic fluid embolism, septic embolism, tumoral embolism, and nonthrombotic pulmonary artery embolism should be considered, especially when anticoagulation treatment fails [2].

Intravascular leiomyomatosis (IVL) is a rare condition, up until now only described in case reports. It is characterized by the growth of benign smooth muscle cells within vascular structures without invading the tissue. In the majority of cases, the neoplasm originates from the uterus, with most common extrauterine locations being the pelvic and broad ligament vessels, and more rarely, the inferior vena cava up to the pulmonary vasculature [3]. Tumors can emerge from the media of peripheral veins, by metastatic spread, or grow into the pelvic veins by direct invasion [4]. IVL with cardiac extension was first published in an autopsy report in 1907 by Durck [5].

Symptoms of IVL depend on the site of origin and the extent of intravascular invasion. In its most severe form, the tumor will migrate and further develop in the pulmonary artery which may cause symptoms of dyspnea, chest pain, and syncope.

The diagnosis of IVL as a cause of nonthrombotic pulmonary embolism is made by a multidisciplinary approach. Primarily, a comprehensive study of the past medical history must be obtained as this condition is often seen in middle-aged women with a history of hysterectomy for a fibromatous uterus [6]. Equally, anamnesis, clinical examination, and imaging by CT with contrast medium enhancement or T1-weighted magnetic resonance images (MRIs) are required [7, 8]. CT with contrast medium enhancement typically shows a hypoattenuating intravascular filling defect. In combination with a fibromatous uterus on MRI, this may suggest IVL [2], although the presence of a normal uterus on imaging does not exclude it [3]. Echocardiography is used to evaluate intracardiac extension, repercussions on the right heart function, and differential diagnosis of other intracardiac masses [9]. To differentiate from a malignant tumor, a preoperative diagnostic biopsy can be obtained [10], e.g., by right heart catheterization as was done in our case. Macroscopically, the tumor has a “wormlike” appearance. On histological and immunohistochemical examination, the tumor will show benign-appearing smooth muscle cells (desmin and alpha SMA positivity) with a weak to strong expression of estrogen receptor (ER) and progesterone receptor (PR) [3, 11].

Complete surgical removal of the intravascular tumor is recommended to relieve symptoms and prevent possible life-threatening complications depending on the location of the IVL [4]. Hysterectomy with bilateral salpingo-oophorectomy is also favored to prevent recurrence [12]. Hormonal therapy is most commonly used in patients unfit to undergo surgical treatment, in patients with incomplete resection or as neoadjuvant therapy to surgery. Tamoxifen, medroxyprogesterone, and gonadotropin-releasing hormone (GnRH) agonists in premenopausal women may be used. Results, however, are diverse ranging from minimal to meaningful response [9]. In our case, the gynecologist decided not to perform a hysterectomy since the risk of relapse was considered low due to the postmenopausal state of the patient and there was no strong evidence of important intracaval tumor thrombosis. Recurrence in patients who were treated by complete, surgical resection appears to be rare. To detect residual tumor growth or relapse, follow-up by six monthly to annual CT or MRI is advised [9, 13].

## 4. Conclusion

Intravascular leiomyomatosis with extension to the pulmonary arteries is a very rare condition. In case of unresolved pulmonary embolism, nonthrombotic causes, like an intima sarcoma or intravascular leiomyomatosis, should be considered.
